# Machine learning algorithms for mode-of-action classification in toxicity assessment

**DOI:** 10.1186/s13040-016-0098-0

**Published:** 2016-05-13

**Authors:** Yile Zhang, Yau Shu Wong, Jian Deng, Cristina Anton, Stephan Gabos, Weiping Zhang, Dorothy Yu Huang, Can Jin

**Affiliations:** Department of Mathematical and Statistical Science, University of Alberta, T6G 2G1, Edmonton, Canada; Department of Mathematics and Statistics, Grant MacEwan University, T5P 2P7, Edmonton, Canada; Department of Laboratory Medicine and Pathology, University of Alberta, T6G 2B7, Edmonton, Canada; Alberta Health, T5J 1S6, Edmonton, Canada; Alberta Centre for Toxicology, University of Calgary, T2N 4N1, Calgary, Canada; AACEA Biosciences Inc, San Diego, 92121 USA

**Keywords:** Time-concentrations response curve, Mode of action, Wavelet transform, Dose response curve, Machine learning, Support vector machine, Artificial neural network

## Abstract

**Background:**

Real Time Cell Analysis (RTCA) technology is used to monitor cellular changes continuously over the entire exposure period. Combining with different testing concentrations, the profiles have potential in probing the mode of action (MOA) of the testing substances.

**Results:**

In this paper, we present machine learning approaches for MOA assessment. Computational tools based on artificial neural network (ANN) and support vector machine (SVM) are developed to analyze the time-concentration response curves (TCRCs) of human cell lines responding to tested chemicals. The techniques are capable of learning data from given TCRCs with known MOA information and then making MOA classification for the unknown toxicity. A novel data processing step based on wavelet transform is introduced to extract important features from the original TCRC data. From the dose response curves, time interval leading to higher classification success rate can be selected as input to enhance the performance of the machine learning algorithm. This is particularly helpful when handling cases with limited and imbalanced data. The validation of the proposed method is demonstrated by the supervised learning algorithm applied to the exposure data of HepG2 cell line to 63 chemicals with 11 concentrations in each test case. Classification success rate in the range of 85 to 95 % are obtained using SVM for MOA classification with two clusters to cases up to four clusters.

**Conclusions:**

Wavelet transform is capable of capturing important features of TCRCs for MOA classification. The proposed SVM scheme incorporated with wavelet transform has a great potential for large scale MOA classification and high-through output chemical screening.

**Electronic supplementary material:**

The online version of this article (doi:10.1186/s13040-016-0098-0) contains supplementary material, which is available to authorized users.

## Background

In recent years, considerable progress has been reported in the study of toxicity profiling using in vitro assays [1]. It is important to develop fast and effective methods capable of analyzing large amount of in vitro data set [2, 3]. By comparing the response profiles of chemicals with known mode of actions (MOAs), we are able to infer the MOA of tested chemicals [4, 5]. One such in vitro assay utilizes the real-time cell analysis system (RTCA) [6–8]. The RTCA system integrates the micro-electrode on the bottom of the wells, such that the electronic impedance data reflect adherent cells status including cell number, cell morphology and adhesion strength. The impedance data at different time points are measured and converted to the cell index (CI) data for further analysis [9, 10]. The system allows multi-concentration assays, such that the Time Concentration Response Curves (TCRCs) can be generated and recorded. The TCRC profiles provide useful information to study the cell-chemical interaction mechanism.

A few analysis methods have been developed to extract useful information from the TCRCs. For example, LC_50_ reflects the chemical concentration that leading to killing 50 % of tested cells [11], KC_50_ uses an exponential model to calculate the LC_50_ value [11–13], AUC_50_ represents the area under the normalized TCRCs, which can be employed to evaluate the toxicity [14]. Based on these indices, further classification or pattern recognition can be investigated. However, these indices only provide partial information of TCRCs and some significant features may not be uncovered. All these indices have the primary goal of detecting toxicity potency of the testing chemicals. The application into MOA classification is indirect, and not tested [15, 16].

MOA describes a cellular level functional change, which is a result of exposure of a living organism to a chemical. According to a pre-set criterion, the chemicals can be classified into different MOA clusters [17]. The machine learning approach has already been utilized in life science research including toxicity classifications [18, 19], analyzing high throughput screening data [20], and drug design [21]. Cheng et al. [22] investigated the toxicity pattern recognition for diverse industrial chemicals with substructure. Vanneschi et al. [23] compares different machine learning algorithms in classifying patients by using breast cancer dataset. Recently, Beck et al. [24] investigate the machine learning by random forests and logistic regression classifiers in bacterial vaginosis (BV) classification, Lareau et al. [25] apply machine learning to analyze functional effectors in microarray data, Lu et al. [26] compares four supervised learning methods in modeling the differentiation of CD4+ T cell.

In this study, we focus on MOA classification for the 63 chemical compounds screening data provided by the Alberta Centre for Toxicology. The list of the chemicals and their ten-cluster MOA classification are given in Additional file 1. The same chemicals were investigated by Pan et al. [13, 14] and Xi et al. [27] for toxicity assessment. Instead of using end-point results, the goal of this study is to develop a new machine learning methodology utilizing the entire TCRCs data recorded for the 63 chemical compounds to perform MOA clustering analysis. The results were validated with the known MOA classification.

**Fig. 1:**
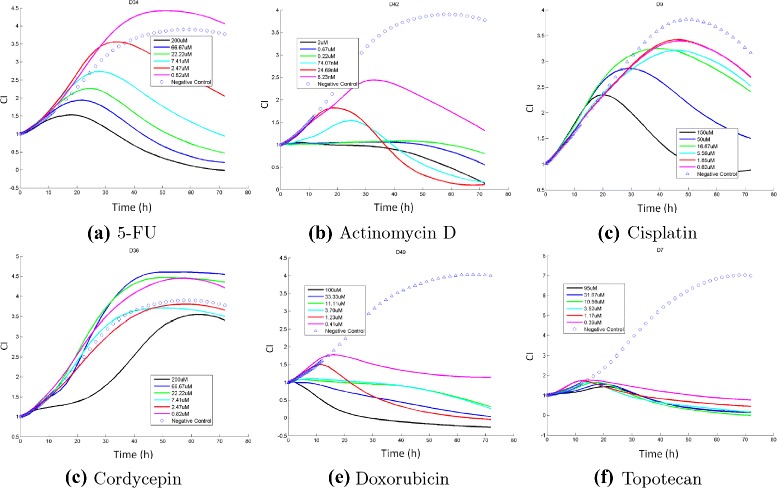
TCRCs from Cluster 1: DNA/RNA-Nucleic acid target. The detail of chemicals in (**a**)-(**f**) are provided in the Additional file 1

**Fig. 2:**
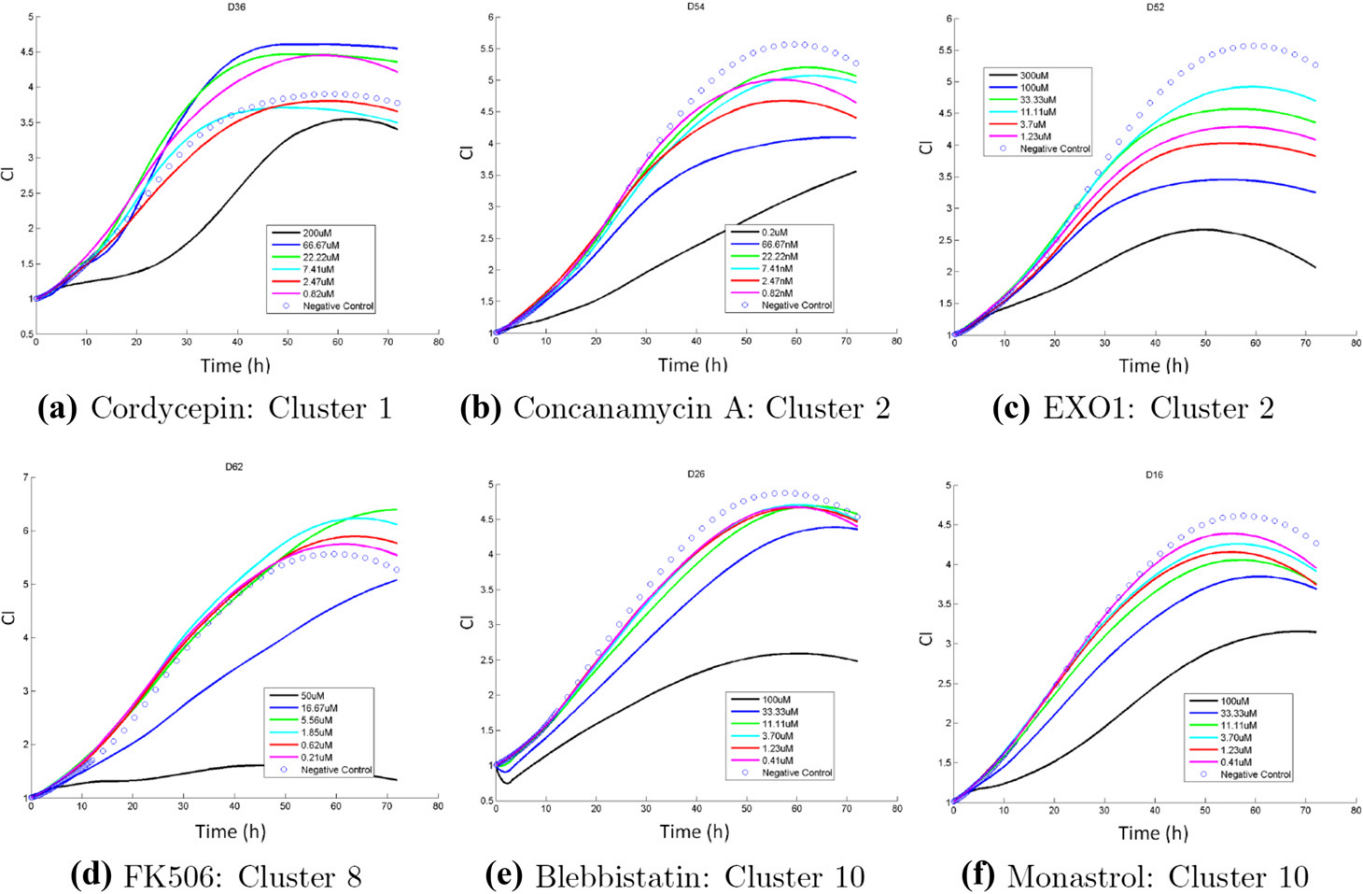
TCRCs from four different clusters. The detail of chemicals in (**a**)-(**f**) are provided in the Additional file 1

It should be noted that it is not trivial to uncover the MOA correlation from the TCRC profiles. In Fig. 1, we display the TCRC profiles of six compounds in cluster 1: DNA/RNA- Nucleic Acid Targets, and the corresponding TCRCs are quite different. However, compounds with different clusters may have similar profiles as illustrated in Fig. 2 showing TCRCs from four different clusters resemble with each other. When the concentration is small, the TCRC profile is very close to the negative control curve. Therefore, to present a better illustration, only the six highest concentrations and the negative control are plotted in Figs. 1 and 2.

The main contributions presented in this work are twofold. First, a novel computational tool is developed based on machine learning for toxicity assessment which was validated for the effectiveness using the TCRCs of 63 chemicals with known MOAs as input. The machine learning methods are based on the artificial neural network (ANN) and support vector machine (SVM) with supervised learning algorithm. Second, wavelet transform is implemented to resolve the difficulty due to taking a large data set from the entire TCRCs. Therefore, instead of directly using the TCRCs as input data to the machine learning algorithms, the wavelet coefficients are selected as input. The application of wavelet preprocessing step not only significantly reduces in the input data, but extracts useful information and features of the original TCRCs. Consequently, success rate in clustering analysis is improved.

The remainder of this paper is organized as follows. The materials and data preprocessing of the present study is first previewed. The next section focuses on the methods based on machine learning approach using ANN and SVM, and the application of wavelet transform is discussed. To validate the developed computational tools, we present binary and multi-cluster classifications applied to the 63 compounds in the fourth section. The effectiveness of SVM is demonstrated by the excellent agreement resulted from the known clustering based on MOA applied to the tested chemical compounds. The use of DRCs is proposed, and the advantage of utilizing DRCs to enhance the performance of the machine learning algorithm for limited data set is reported. Finally, conclusion remark is presented.

## Materials and data preprocessing

### Cell line

Human hepato carcinoma cells line-HepG2 (ATCC, cat. no. HB-8065) were grown and tested in EMEM basal media supplemented with 10 % fetal bovine serum. All growth and assay were conducted in 37 °C tissue culture hood with 95 % humidity and 5 % CO2.

### Chemicals

All testing chemicals were at least 95 % purity. They were obtained through commercial sources including Sigma-Aldrich, Cayman Chemicals, Tocris, and Santa cruz biotechnologies. Three solvents were used for powder solubilization: water, DMSO or ethanol. The solvent providing highest solubility when diluted in assay media were used for stock solution preparation. Stock solution were aliquoted for single usage and stored at –20 °C. The highest testing concentration is at most 1/500th of the stock concentration, so that solvent (DMSO or ethanol) concentration are no more than 0.2 %. Each chemical were tested with 11 concentrations, with 1:3 serial dilution.

### RTCA HT assay

The xCELLigence RTCA HT system developed at ACEA Biosciences Inc. runs four 384x well E-Plates on four independent HT Stations. The continuous cell monitoring enabled both transient and long term effects being recorded. The system was integrated with the Biomek FXp System and the Cytomat hotels for fully automated liquid handling and plate shuffles. The HepG2 cells were seeded into the E-plate 384, and monitored once an hour in the first 24 h for initial attachment and growth. 11 concentrations of each chemical were applied into the wells by using automatic pipetting. The cellular responses were continuously monitored for at least 72 h.

### Data preprocessing

The RTCA technology monitors the impedance signal generated by cells covering electrodes. The impedance signal R is converted to a parameter Cell Index (CI) with the following formulation [28, 29]:

1 $$\begin{array}{@{}rcl@{}} CI = \max_{k=1,\cdots,K}\left[\frac{R_{cell}(\,f_{k})}{R_{b}(\,f_{k})}-1\right], \end{array} $$

where $R_{cell}\left (\,f_{k}\right)$ and $R_{b}\left (\,f_{k}\right)$ are the electrode impedance with and without cell in the well, and *k* is the discrete time points.

To focus on cellular response to testing chemicals, *CI* differences from seeding and growth variation were minimized by using Normalized Cell Index (NCI), which is given by

2 $$\begin{array}{@{}rcl@{}} NCI[k] = \frac{CI[k]}{CI[0]},~k=1,2,\cdots, K. \end{array} $$

Here, k refers to different time points after testing chemical addition, and k =0 refers to the time point right before treatment.

Because not much information can be extracted from the TCRCs before adding the compounds, we focus on the NCI data after chemical treatment. Moreover, for the irregular data set, the time grids for different compounds are not uniform. We apply a cubic spline to interpolate the non-uniform data into uniform grids, where the time interval is one hour for the interpolated data set. The uniform data set enables the use of wavelet transform, which is critical in data reduction and better extracting the features from the original TCRCs data set.

## Methods

Two machine learning algorithms, namely artificial neural network (ANN) and support vector machine (SVM) were used in this study. The application of wavelet transform to enhance the performance and effectiveness of ANN and SVM will also be introduced.

### Artificial neural network

**Fig. 3:**
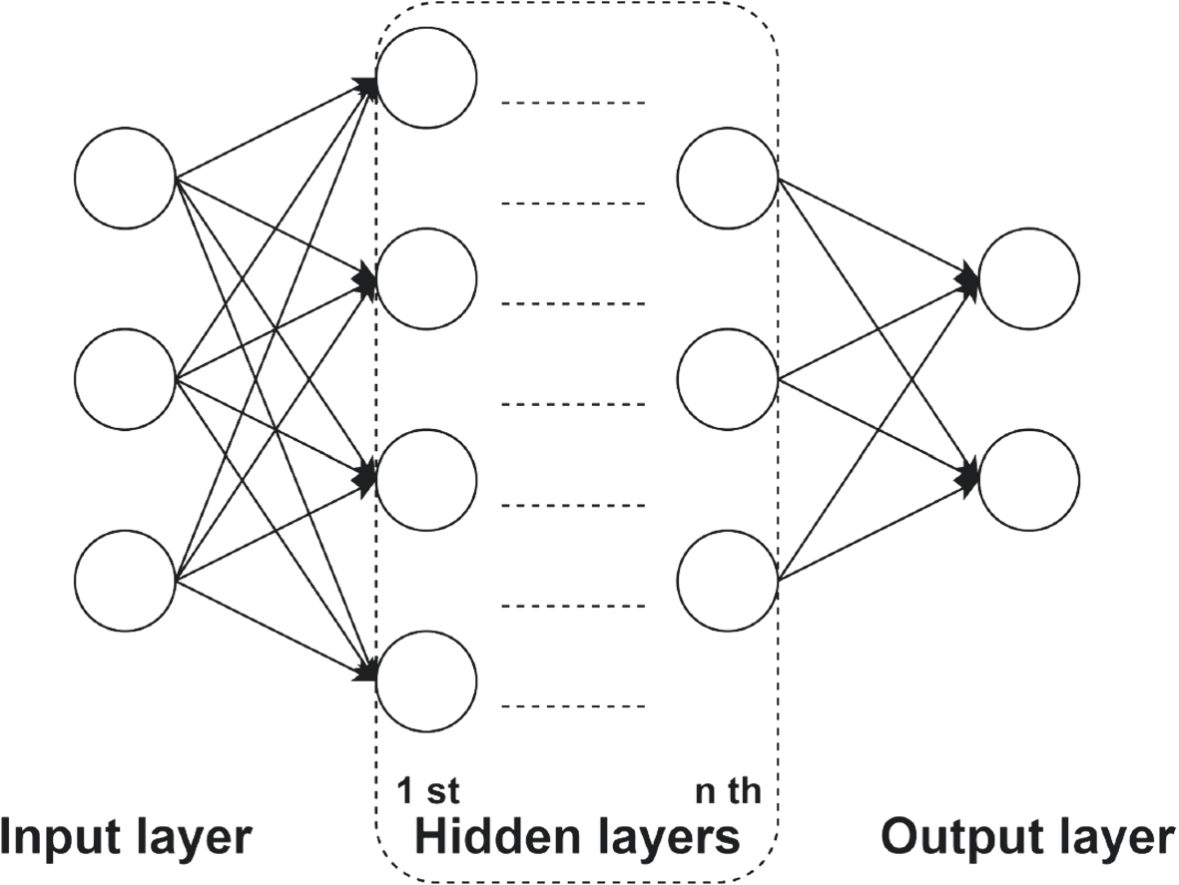
Feedforward *n*-layer ANN

Artificial neural network (ANN) is inspired by a biological neural network, and it can be considered as a computational information processing model simulating a “brain like" system of interconnected processing units. ANN has already been applied in toxicity study [30–32]. A typical feedforward multi-layer ANN [33] is shown in Fig. 3.

In the network, there are one input layer and one output layer. The number of input neurons equals to the number of attributes, and the number of output neurons depends on the particular application of the network. In the present study, input neurons are given by the time series of TCRCs, and the output neurons are determined by the number of clusters being classified. The layers between input and xoutput layers are the hidden layers. The network architecture (i.e., the size of hidden layers and the number of neurons in each hidden layer) is depended upon the complexity of a specified problem under investigation. Each neuron is interconnected with other neurone in the next layer, and the information passing though the neurons are determined by the weights. The weights are computed by a supervised algorithm by presenting the TCRCs data as input with known MOA clustering as desired output in the training phase. The weights will then be adjusted by minimizing a given objective function. The training process is conducted repeatedly until the network achieved a prescribed success rate for all training data and it will then be used to classify the future compounds with unknown MOA. Once the training is completed, the network is capable of performing a specified task rapidly with little computing time and it is particularly suitable for a real time application.

Mathematically speaking, training the ANN is to seek a function *f*: *X*$\rightarrow $*Y* to fit a set of example pairs (*x*,*y*), x ∈ X, y ∈ Y. The network as a whole can be regarded as a multivariate function or multivariate vector function if there is multiple outputs. By minimizing *f*(*X*)−*Y*, we are able to find a function *f* to approximate the relationship between the attribute of sample set *X* and the corresponding cluster *Y*. By inputting the attribute of future sample $\hat {x}$ to the obtained function *f*, its classification information $\hat {y}$ can be inferred. Obviously, the information in the minimization process is unknown; the training process of ANN is actually a black box model. However, since there exists many local minimum in minimizing *f*(*X*)−*Y*, the same training set (*X*,*Y*) may produce totally different network parameters and lead to inconsistent classification results. This is particularly true for the MOA classification, where the data set is relatively small and imbalanced.

### Support vector machine

In addition to ANN, another important machine learning algorithm is the support vector machine (SVM). The application of SVM in toxic predictions has been reported in [34, 35]. As one of the popular classifiers, the idea of SVM is quite different from that of ANN. To perform a classification for a given data set, SVM uses a hyperplane to separate the sample data points [36]. Assuming there is a set of data *x*_*i*_ along with their corresponding label *y*_*i*_, and considering the data is composed of two clusters denoted by -1 and 1, then we have the data space

$$\begin{array}{@{}rcl@{}} D = \{(x_{i},y_{i})|x_{i}\in R^{P}, y_{i}\in \{-1,1\}\}_{i=1}^{n}. \end{array} $$

Initially, we hope to find a hyperplane separating the sample data, in which each class of data belongs to one side. Let the plane be

$$\begin{array}{@{}rcl@{}} w\cdot x-b = 0. \end{array} $$

The problem of constructing such a hyperplane is to ensure its robustness. Supposing that there are two samples very closed to each other but on the different sides of the hyperplane, then it is not reasonable to classify them into different categories. To resolve the problem, we select two hyperplanes such that they separate the data with no point between them. The best robustness is achieved when the distance between them is maximized. The region bounded by the planes is called "margin", and the two hyperplanes can be rewritten as

$$\begin{array}{@{}rcl@{}} w\cdot x-b = \pm 1, \end{array} $$

therefore, the distance between them is defined by $\frac {2}{||w||}$. It is clear that to maximize the distance, we need to minimize the ||*w*||. Consider the fact that if the sample *x*_*i*_ belongs to the first class, then *w*·*x*−*b*>1. Similarly, *w*·*x*−*b*<−1 if it is in the second class. Thus, we can rewrite the classification problem as the following optimization problem

$$\begin{array}{@{}rcl@{}} \min ||w|| \mathrm{~subject~to~} y_{i}(w\cdot x_{i}-b)\leq 1~~\text{for}~ i = 1,\cdots, n. \end{array} $$

The weight vector *w* and the parameter *b* are determined by a supervised learning algorithm similar to ANN. Now, the remaining problem is that for a large amount of data in the data space and due to the highly non-linearity in the sample data, it is not possible to divide them into multiple clusters by hyperplanes. This problem can be resolved by considering a mapping from a lower dimensional space to a high dimensional space using a suitable kernel, so that the data are expected to be separable in the high dimensional space. The selection of the kernel is critical to the success of SVM.

Recent studies indicates that the SVM is more accurate and robust than ANN in the chemical classification [37], and it is capable of handling data set with more complex structure. The SVM algorithm used in this study is based on the standard SVM classifier in MATLAB with a Gaussian kernel. Comparing with ANN, the most significant advantage of SVM is that it has global minima instead of local minima, so that the convergence speed is significantly faster than ANN. Therefore, in the multi-cluster classification, SVM is used as a main tool. Note that the classification of SVM is always binary, but the binary classification algorithm can be recursively applied for applications to multiple clusters. The details will be discussed in the next section.

### Wavelet transform

The training process is a crucial component to ensure the success of a learning machine. To certain extent, large input data in the training will affect the structure of learning machine and also introduce more difficulty in the supervised learning. In the present study, the input data contains the time series of TCRCs, and it could have more than 850 points. For ANN, the size of the hidden layers and the number of neurons depends on the number of input neurons. Therefore, taking a large data set of input is not a trivial task for a learning machine, and this may be the reason why no reference has been reported on using ANN or SVM for toxicity assessment using TCRCs as input. We now propose a novel idea to deal with large input data by using wavelet transform. Different from the standard Fourier transform, which is only localized in frequency, wavelets are localized in both time and frequency. Wavelet transform has been successfully demonstrated to be a powerful tool for data compression and feature extraction in signal and image processing.

Let {*e*_*i*_} be an orthonormal and complete set in a Hilbert space H, and *T* be an arbitrary vector in H [38], then

$$\begin{array}{@{}rcl@{}} T = \sum_{i} <T,e_{i}>e_{i}, \end{array} $$

here *T* is the vector consisting of the data from TCRCs, *e*_*i*_ is the orthonormal basis, <,> is the inner product and <*T*,*e*_*i*_> denotes the coefficients under the basis *e*_*i*_. By selecting a set of orthonormal vectors *e*_*i*_, we can use wavelet coefficients to represent the TCRCs toxicity data. An orthonormal basis *ψ*_*s*,*τ*_(*t*) [39] having scale parameter *s* and translation parameter *τ* can be expressed in the following form:

$$\begin{array}{@{}rcl@{}} \psi_{s,\tau}(t)=\frac{1}{\sqrt{s}}\psi\left(\frac{t-\tau}{s}\right). \end{array} $$

Let *T*(*t*) be the original TCRCs data, then the wavelet coefficients *X*=<*T*,*e*_*i*_> is a function of *s* and *τ* given by

$$\begin{array}{@{}rcl@{}} X(s,\tau) =\int T(t)\psi^{*}_{s,\tau}(t)dt \end{array} $$

where ^∗^ denotes the complex conjugation, this equation shows how a *T*(*t*) is decomposed into a set of wavelet basis function *ψ*_*s*,*τ*_(*t*). Accordingly, *T*(*t*) can be recovered by the inverse wavelet transform as

$$\begin{array}{@{}rcl@{}} T(t) = \int\int X(s,\tau)\psi^{*}_{s,\tau}(t)dsd\tau, \end{array} $$

where the wavelets are generated from one mother wavelet *ψ*(*t*) by scaling and translation.

**Fig. 4:**
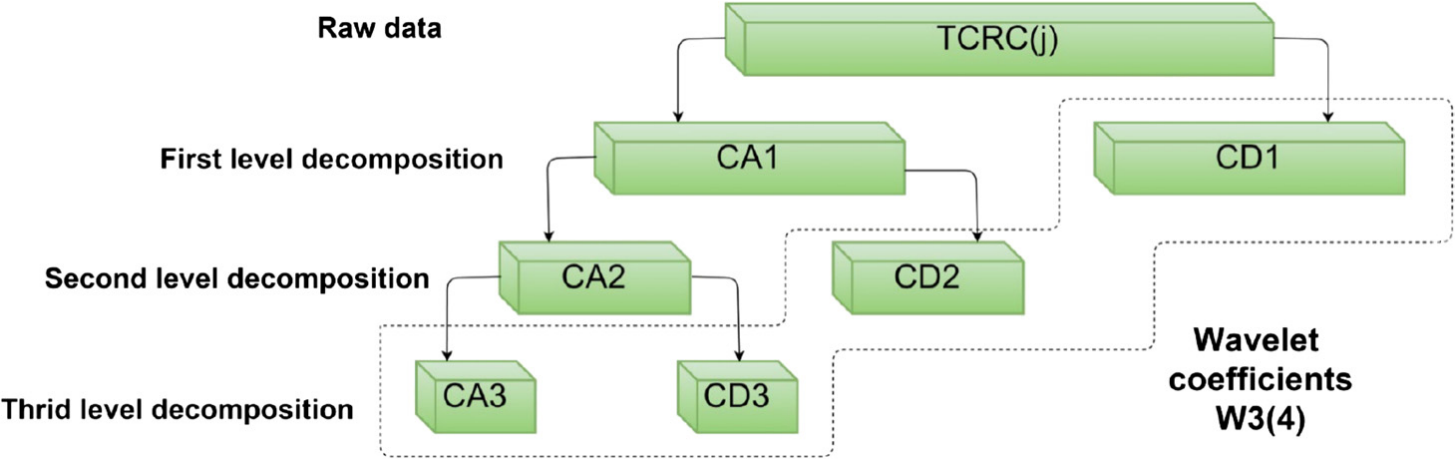
Three-level wavelet decomposition

One of the advantages of wavelet transform lies in its ability to extract multiscale information from the input data. By recursively applying wavelet transforms, it leads to multi-level wavelet decomposition. The procedure for a three-level wavelet decomposition is illustrated in Fig. 4, where the raw TCRCs are represented by *T*. In the first level of wavelet transform, the original signal *T* is decomposed into two vectors CA_1_ and CD_1_ representing the approximate and detail coefficients, respectively. In the second level of decomposition, the wavelet transform is applied again to CA_1_ resulting two decomposition CA_2_ and CD_2_. In a *n*-level wavelet decomposition, the wavelet transform is applied recursively to decompose the approximation coefficient CA _*j*_ at the *j*th level into the coefficients CA _*j*+1_ and CD _*j*+1_. Therefore, applying an *n*th level wavelet decomposition, we have one approximation coefficient CA _*n*_ and detail coefficients CD _*n*_, CD _*n*−1_, ⋯, CD_2_, CD_1_. We now denote all wavelet coefficients at the *n*th level decomposition as W _*n*_. When particular coefficients are used instead of the entire wavelet coefficients, we denote the coefficients as W _*n*_(*m*) where *m* is the number of coefficients. Generally speaking, the selection of wavelet coefficients starts from the approximation coefficient and highest level of detail coefficients, because the detail coefficients at lower level always contain small fluctuations including noise from the original information [40]. Consider a three-level decomposition (i.e., *n*=3), W_3_(4) means that four wavelet coefficients: CA_3_+CD_3_+CD_2_+CD_1_ are kept and W_3_(2) implies taking two wavelet coefficients CA_3_+CD_3_.

**Fig. 5:**
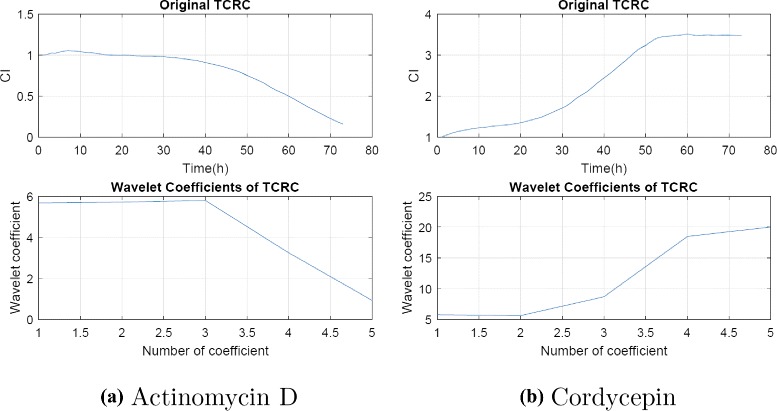
Plots of original TCRCs and wavelet coefficients W_5_(1) for (**a**) Actinomycin D and (**b**) Cordycepin

To demonstrate the capability of extracting important feature of the original data using fewer wavelet coefficients, we apply wavelet transform to two compounds listed in cluster 1. Figure 5 displays the profiles of one concentration TCRC for two different compounds and the corresponding profiles using wavelets W_5_(1). It is clear that the profiles are in good agreement, but a tremendous data reduction over 90 % is achieved using wavelet transform. Note that the original TCRC contains 72 data, while only five wavelet coefficients are in W_5_(1).

**Fig. 6:**
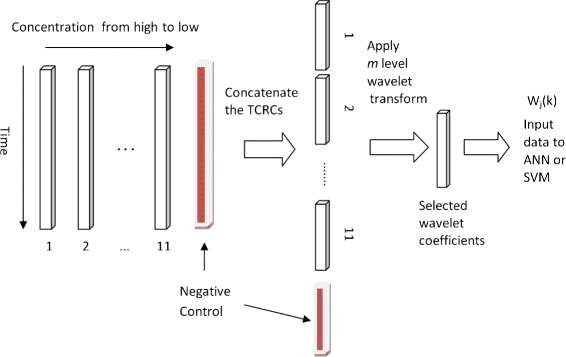
Input data to machine learning using wavelet transform

We now illustrate how to construct input data for machine learning. A given set of TCRCs is arranged as shown in Fig. 6, where 1 denotes the TCRC with the highest concentration, 2 for the next highest concentration, and 11 for the lowest concentration. By concatenating the vectors according to the order 1, 2, ⋯, n, we form a new vector TCRC(*n*). Here, TCRC(1) contains data from the highest concentration, TCRC(2) contains the first two highest concentrations and TCRC(11) contains data from all 11 concentrations. It will be demonstrated later that including the negative control will enhance the performance of the developed machine learning tools. The new vector TCRC(*n*) can now be considered as input data to the machine learning algorithm. However, we also consider using wavelets by applying wavelet transform to TCRC(*n*) and selecting specified multi-level wavelet coefficients as input to ANN or SVM. The advantages of using wavelets will be clearly demonstrated in the next section.

## Results and discussion

**Fig. 7:**
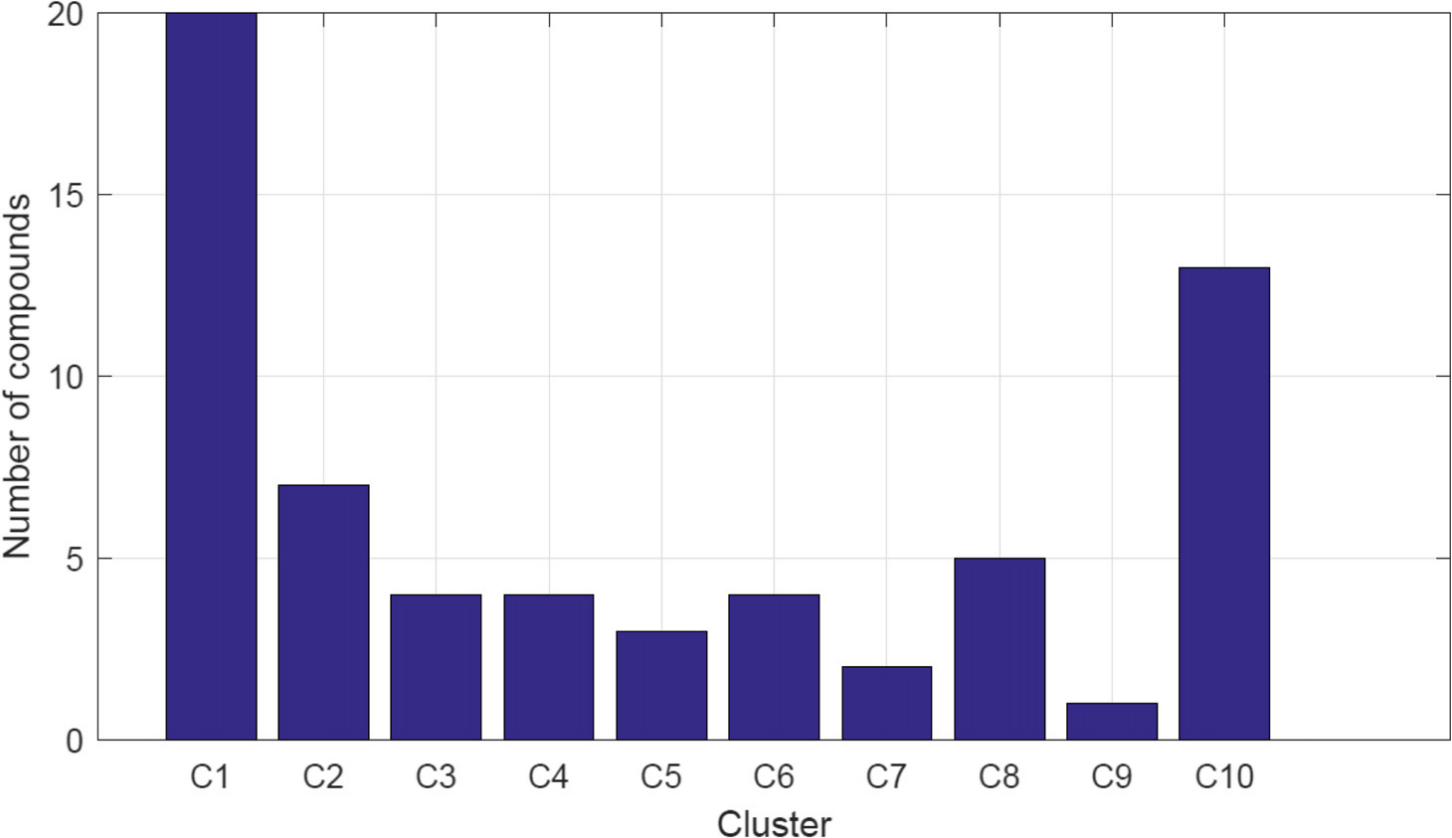
Distribution of 63 compounds

To validate the developed machine learning tools based on ANN and SVM for MOA classification and to verify the effectiveness of using wavelets for input data preprocessing, we present the following computational simulation applied to the 63 compounds. As shown in Appendix, there are 10 clusters in the 63 compounds with imbalanced cluster distribution as illustrated in Fig. 7. Note that C1 and C10 contain 33 compounds, and they make up more than half of the 63 compounds. Here, we will not consider the three clusters C5, C7 and C9, since each cluster comprises only 3, 2 and 1 compounds, respectively.

For the ANN, a feedforward three-layer network with 24−12−6 neurons in the hidden layers is used. The results are not sensitive even by doubling the hidden-layer neurons. In the training process, the network is accepted when the success rate of the targeted classification reaches 85 %. For problems with limited and imbalanced data, setting a higher success rate for training may lead to over-fitting and producing an inferior network performance.

### Binary classification

We first consider the classification for the two largest clusters, namely C1 with target class DNA/RNA and C10 with target class protein. There are 20 compounds in C1 and 13 compounds in C10, therefore, using 70 % training data implies that 14 compounds in C1 and 9 compounds in C10 are available as training set. The remaining 30 % data, 6 compounds in C1 and 4 compounds in C10 will be considered as test set. All simulation reported in this work are based on 70 % training data and 30 % for the testing data.

We define the success rate (SR) for the classification as

$$\begin{array}{@{}rcl@{}} SR = \frac{\textrm{Number of compounds classified into correct MOA}}{\textrm{Total number of compounds in datasets}}. \end{array} $$

Once the number of compounds in the training set is determined, the developed machine learning tools can be used to perform the classification for C1 and C10. The effectiveness of ANN and SVM can then be evaluated by the computed success rate (SR). For example, in the case of 70 % training set, there are 10 compounds available for the test data. If 9 of them are classified into the correct clusters in C1 or C10, then the successful rate is 90 %. However, it is not reasonable to conclude about the performance of the classifier merely based on one result, especially because the current problem has limited test data for some clusters. To obtain a reliable conclusion for the machine learning tools, the classification process is conducted 100 times, and the training and test set are randomly selected for each simulation. Consequently, 100 SR will be computed from the 100 classifications using 100 different partitions of training and test set. The overall average of the 100 SR will be recorded as the final success rate. Different from the conventional cross validation, which is based on a fixed partition of the data set, the data set partition in the present study is in a more random fashion. This is due to the limited size of the data available in this study, so that a fixed partition can cause significant bias in the classification SR.

As mentioned before, the performance of the machine learning algorithms will be affected by the input data. Intuitively, one may expect that feeding more information to the input should improve the performance for the machine learning tools. In this study, the input is given by the TCRCs and a typical data set consists of 11 concentrations. Let TCRC(1) denote the data taking only the highest concentration, TCRC(2) for data with the first two highest concentrations and TCRC(11) for data including all 11 concentrations. In Tables 1 and 2, we report the SR for ANN and SVM using 70 % of the observations as training data. TCRC(*j*) with *j*=1,2,⋯,11 denotes input using the raw data, and W _*i*_ for *i*=1,2,..,5 indicates the corresponding wavelet coefficients from the *ith*-level wavelet decomposition is taken as input data. Using the raw data TCRC(*j*), the SR is poor and unacceptable when *j*=1. As expected, the SR for ANN is improving when the value of j is increased. However, it is observed that the SR for SVM with TCRC(*j*), *j*=3,4,⋯,7 is even lower than the SR using the highest concentrations data TCRC(1)and the first two highest concentrations TCRC(2). The advantage of using wavelet coefficients W _*i*_ instead of the TCRC(*j*) raw data is clearly demonstrated from the results presented in in Tables 1 and 2. By first applying the wavelet transform to TCRC(*j*) data, consistent improvement in the SR results for both ANN and SVM is achieved as more data are taken as input. Using only the highest concentration TCRC(1), the ANN SR is improved by 35 % when the input data is using wavelet coefficients instead of the raw data. In addition to confirming that wavelet coefficients capture all features in the raw TCRCs data and yield better SR for ANN and SVM, another important enhancement can be achieved by selecting appropriate wavelet coefficients such that much less input data is needed for the machine learning tools. The details and the discussion will be presented shortly.

**Table 1:** ANN SR with different concentrations

	Raw	W_1_	W_2_	W_3_	W_4_	W_5_
TCRC(1)	0.550	0.705	0.738	0.731	0.742	0.701
TCRC(2)	0.711	0.782	0.760	0.774	0.750	0.795
TCRC(3)	0.741	0.782	0.779	0.774	0.798	0.787
TCRC(4)	0.739	0.788	0.811	0.796	0.817	0.820
TCRC(5)	0.750	0.803	0.802	0.829	0.822	0.811
TCRC(6)	0.767	0.819	0.836	0.817	0.826	0.827
TCRC(7)	0.770	0.831	0.825	0.850	0.843	0.817
TCRC(8)	0.838	0.856	0.836	0.861	0.836	0.832
TCRC(9)	0.864	0.852	0.845	0.873	0.829	0.827
TCRC(10)	0.859	0.871	0.849	0.830	0.834	0.838
TCRC(11)	0.855	0.879	0.865	0.863	0.861	0.855

**Table 2:** SVM SR with different concentrations

	Raw	W_1_	W_2_	W_3_	W_4_	W_5_
TCRC(1)	0.690	0.667	0.698	0.688	0.705	0.669
TCRC(2)	0.746	0.766	0.742	0.744	0.727	0.764
TCRC(3)	0.694	0.802	0.789	0.785	0.787	0.774
TCRC(4)	0.664	0.817	0.834	0.812	0.837	0.838
TCRC(5)	0.627	0.846	0.836	0.851	0.857	0.838
TCRC(6)	0.636	0.870	0.878	0.853	0.864	0.866
TCRC(7)	0.634	0.849	0.852	0.874	0.870	0.846
TCRC(8)	0.749	0.894	0.869	0.876	0.867	0.875
TCRC(9)	0.789	0.867	0.867	0.908	0.868	0.881
TCRC(10)	0.788	0.880	0.853	0.859	0.866	0.869
TCRC(11)	0.821	0.888	0.898	0.898	0.890	0.907

**Fig. 8:**
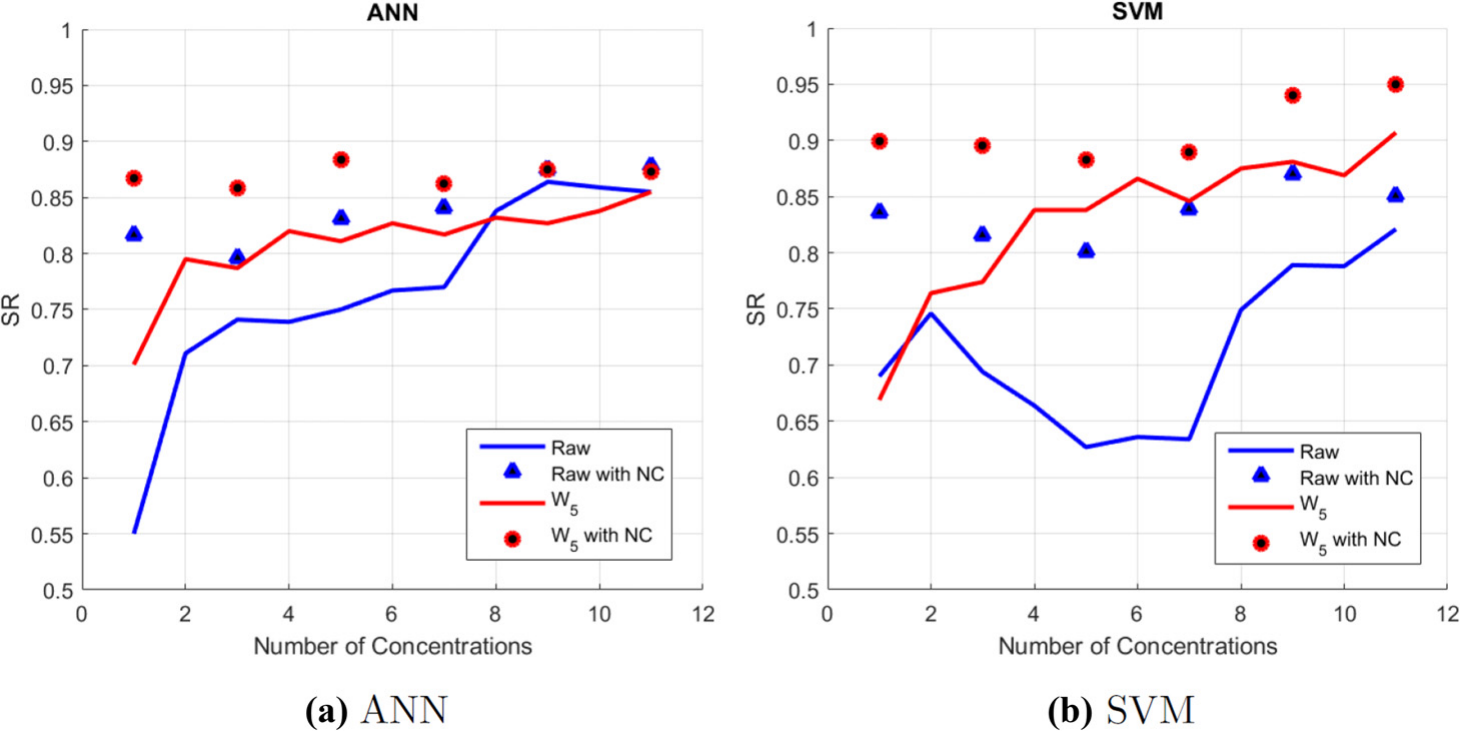
Classification SR of (**a**) ANN and (**b**) SVM by using different number of concentrations

The computational results presented so far are based on input data taken from TCRC(*j*). However, the performance can be further enhanced by taking account information from the negative control (NC). The improvement is due to the NC data containing information of the assays such as the cell plate condition, environment temperature, and so on. Figure 8 illustrates the classification SR for C1 and C10 using machine learning algorithms with input data given by TCRC(*j*) and W_5_ with and without information of NC. There is no doubt that incorporating NC into the input data does play an important role of providing more information to the machine learning algorithms, and this leads to a significant enhancement for ANN and SVM. It is particularly noted that tremendous increase in SR is observed when the input is based on a few TCRC data. Using wavelet transform and the highest concentration TCRC(1) data without and with NC, the SR increases from 0.550 to 0.870 for ANN, and 0.691 to 0.907 for SVM. Among the two data mining tools, it is preferable to use SVM since it consistently produces a higher SR than that using ANN. Hence, the remaining results presented in this work will be based on SVM and with TCRC(*j*) including negative curve as input data.

By including the negative control (NC) with the TCRC(11), the input contains 876 data points, and almost the same amount of data will be required for wavelets W _*i*_, *i*=1,2,⋯,5 if all coefficients in wavelet decompositions are kept. However, it is well known that wavelet transform is especially effective for data compression. Utilizing this attractive feature, we could achieve the same or better performance by appropriately pruning the wavelet coefficients. Consequently, much less data is needed as input for SVM. Now, consider a 5-level wavelet decomposition is applied to TCRC(11) with NC, and let W _5_(*i*) denote the corresponding wavelet coefficients, where *i*=1,2,⋯,6. Note that W _5_(6) corresponds to the case when all the wavelet coefficients are included, i.e., W_5_(6): CA_5_ + CD_5_ + CD_4_ + CD_3_+ CD_2_ + CD_1_ and only one set of coefficients is kept in W _5_(1), where W _5_(1) is the CA_5_. The length of input data for TCRC(11) with NC and W_5_(i) are listed in Table 3. Compared with the original TCRC(11) data with NC, savings of 74 % and 97 % are achieved when using W_5_(4) and W_5_(1) as input. Clearly, a tremendous data reduction is achieved by pruning the wavelet coefficients. Applying the same approach for the highest concentration data TCRC(1) with NC, the corresponding wavelet coefficients W _5_(*i*) are shown in Table 3.

**Table 3:** Length of input data using raw data and wavelet coefficients

	Raw	*W*_5_(6)	*W*_5_(5)	*W*_5_(4)	*W*_5_(3)	*W*_5_(2)	*W*_5_(1)
TCRC(11) + NC	876	889	450	229	117	60	30
TCRC(1) + NC	146	157	83	45	25	14	7

Recall that by taking all wavelet coefficients from TCRC(1) and TCRC(11), the SVM SR shown in Fig. 8 is 90 % and 95 %, respectively. In Table 4, we evaluate the SVM performance for C1-C10 classification using coefficients based on various wavelet decomposition levels as listed in Table 3. Using the information from all TCRC(11) data, the SR is the range of 80.8 % to 96.4 %. It is remarkable to observe that even using W _5_(2) and W _5_(1) with 60 and 30 data points as input, over 80 % SR is achieved. Note that the original raw data contains 876 data, and using W _5_(2) and W _5_(1), the input data is being reduced by 93 % and 97 %. It is also worthwhile to note that over 90 % SVM SR is recorded when the input data is based on only the highest concentration TCRC(1) and with W _5_(*i*) for *i*>3. Without going through a detail study to optimize the wavelet decompositions, we now fucus on the data mining tools based on SVM(11) and SVM(1). Here, SVM(11) denotes SVM using input data from the W _5_(4) based on entire TCRC(11), and SVM(1) corresponds to input using W _5_(6) from the highest concentration TCRC(1). Thus, the input data in SVM(11) and SVM(1) are 229 and 157, and this produces a reduction of 74 % and 82 % compared to taking entire raw data TCRCs with 11 concentrations.

**Table 4:** SVM SR for C1 and C10 classification

	Raw	*W*_5_(6)	*W*_5_(5)	*W*_5_(4)	*W*_5_(3)	*W*_5_(2)	*W*_5_(1)
TCRC(11) + NC	0.857	0.947	0.944	0.964	0.905	0.821	0.808
TCRC(1) + NC	0.845	0.909	0.904	0.930	0.795	0.779	0.770

In Table 5, we present the two-cluster MOA classification results using SVM(11) and SVM(1). The two-cluster is defined by clustering C1 and C*j* where *j* ≠ 1. Let the error in each classification be (1-SR), and define the average error as E =[(1−*SR*(*C*1/*C*2))+(1−*SR*(*C*1/*C*3))+…+(1−(*SR*(*C*1/*C*10))]/6. The results presented in Table 5 reveal that the performance for SVM(11) and SVM(1) are comparable, and the average error in SR is 0.1592 and 0.1547 for SVM(11) and SVM(1), respectively. However, it is important to note that while SVM(11) produces low SR 74.2 % and 77.9 % for (C1/C2) and (C1/C6) classification, the corresponding SR using SVM(1) increases to 87.9 % and 83.3 %. Therefore, by examining the SR values resulting from SVM(11) and SVM(1), we can enhance the accuracy for the MOA classification. Let SVM denote by selecting the best SR from SVM (1) and SVM(11), and the SVM SR for the two-cluster classification is reported in Table 5. Note that, the average error E for SVM is now reduced to 0.1085. Although further improvement is possible by investigating other data from TCRC(k) where k ≠ 1 and 11 and by optimizing the wavelet coefficients, we will only carry out computation using SVM(1) and SVM(11) and the best value will be recorded as SVM in this study.

**Table 5:** SVM SR for two-cluster classification

	(C1/C2)	(C1/C3)	(C1/C4)	(C1/C6)	(C1/C8)	(C1/C10)
SVM(11)	0.742	0.995	0.845	0.779	0.720	0.964
SVM(1)	0.879	1.000	0.694	0.941	0.657	0.901
SVM	0.879	1.000	0.845	0.941	0.720	0.964

### Multi-cluster classification

In many applications, a data set may contain more than two clusters. Therefore, it is necessary to expand machine learning algorithm from binary classification to multi-cluster classification. ANN can easily be adapted to deal with multi-cluster cases, and we only need to assign the number of output neurons equal to the number of clusters. Since the performance of ANN is not as effective as SVM, we will not present the results using ANN. To carry out multi-cluster classification for SVM, we utilize a tree structure strategy [41]. Due to the imbalanced data in the 63 chemical compounds, our study will focus on extending the SVM algorithm for classifications with three and four clusters.

**Fig. 9:**
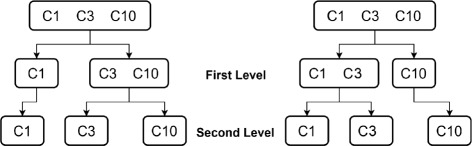
Tree-structure for three-cluster classification

First, consider an example of a three clusters C1, C3 and C10. Since C3 contains only four data sets which is much smaller than C1 and C10, a reasonable tree structure for classification is shown in Fig. 9, in which a binary classification is conducted at each level. Figure 9 illustrates extending a two-level tree structure methodology for three-cluster classification. For the left configuration, we first label both compounds in C3 and C10 as one class C, then a binary classification for C1 and C is carried out. In the second level, the cluster C is further classified into C3 and C10 by using binary classification again. Similarly, for the right configuration, C1 and C3 are first labelled as one class C in the first level, and then be classified in SVM algorithm. Although it is feasible to have a tree structure by first combing C1 and C10 into one class, this selection will not be recommended. It is known that SVM works well for balanced data set such that the training and test data in both groups are almost equal. For the structure given by [C3 and (C1+C10)] with 70 % training, we have a highly imbalanced data since there are only 3 training data in one group and 23 data in the other group. Using the same approach, we consider another three-cluster for C1, C2 and C10. The MOA classification results for the two test cases are shown in Table 6. Obviously, the clustering SR is sensitive to the specified tree structure. The overall SR for [(C1 + C3) and C10] is significantly higher than for [C1 and (C3 + C10)] as reported. However, for the second example, [C1 and (C2 + C10)] will be a better choice.

**Table 6:** SVM SR for three-cluster classification

C ^∗^	C1 and (C10 + C ^∗^)	(C1 + C ^∗^) and C10
C3	0.841	0.968
C2	0.880	0.810

**Fig. 10:**
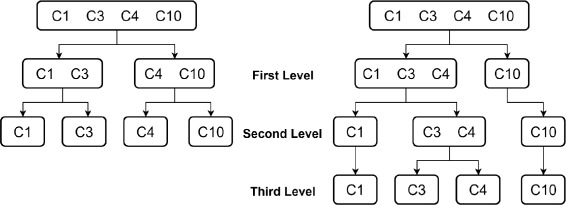
Tree-structure for Four-cluster classification

The methodology using a tree structure approach can be further extended to deal with four-cluster classification, and let consider MOA classification for C1, C3, C4 and C10 as shown in Fig. 10. Note than C3 and C4 contains less data than C1 and C10. By the same argument presented for a three-cluster classification, we propose two-level and three-level configurations for the four-cluster MOA classification. To evaluate the robustness of SVM for multi-cluster classification, we construct another test case by replacing C4 data sets by C2 data. The SVM SR for the four configurations are reported in Table 7. The best configuration is based on [(C1+C*)+C3] and [C10], for which 85.6 % and 84.7 % classification SR is achieved for C^∗^=C4 and C^∗^=C2, respectively.

**Table 7:** SVM SR for four-cluster classification

	Two-level approach		Three-level approach	
C*	[C1+C3]&	[C1+C ^∗^]	[(C1+C ^∗^)+C3]	[(C1+C3)+C ^∗^]
	&[C ^∗^+C10]	&[C3+C10]	&[C10]	&[C10]
C4	0.807	0.750	0.856	0.838
C2	0.839	0.805	0.847	0.825

### Dose response curves

In order to deal with the limited data sets for some clusters considered in this study, we proposed to construct the Dose Response Curves (DRCs), and utilizing the information from DRCs as input to the developed learning algorithm.

Instead of using TCRCs as input data, we now utilize information from Dose Response Curves (DRCs) as input to SVM. The DRCs reveals the effect of the chemicals at different concentrations, and it can be computed from the difference between the time concentration response curves and the negative control curve at a particular time point. Let, denote

3 $$\begin{array}{@{}rcl@{}} {TE}_{t}(k) = \frac{{TCRC}_{t}(k)-{NC}_{t}}{{NC}_{t}}*100 \% \end{array} $$

where *TE*_*t*_(*k*) is the toxicity effect (*TE*) of the chemical with *k*th concentration at time *t*, *NC*_*t*_ is the cell index value of the negative control at time *t*. From this definition, it is clear that when *TE*_*t*_(*k*)=0, it implies that the chemical compound with concentration *k* has no toxicity effect to the cell growth at time *t*. Similarly, we can also define the *TE* by the area under the curve (AUC) as suggested in [14]:

4 $$\begin{array}{@{}rcl@{}} {TE}_{t}(k) = \frac{AUC\{{TCRC}_{t}(k)\}-AUC\{{NC}_{t}\}}{AUC\{{NC}_{t}\}}*100 \% \end{array} $$

where *AUC*{*TCRC*_*t*_(*k*)} denotes the area under the curve TCRC(k) between 0 to *t* hours, *AUC*{*NC*_*t*_} is the area under the negative control curve between 0 to *t* hours. Using *TE*_*t*_(*k*), we can construct a sequence of *TE* at time *t*. For example, using the 11 concentrations TCRCs of the tested 63-chemicals data, the DRC can be computed at time *T* such that

5 $$\begin{array}{@{}rcl@{}} DRC(T) = \left[{TE}_{T}(1) ~~~{TE}_{T}(2) ~~~\cdots ~~~{TE}_{T}(11)\right]. \end{array} $$

**Fig. 11:**
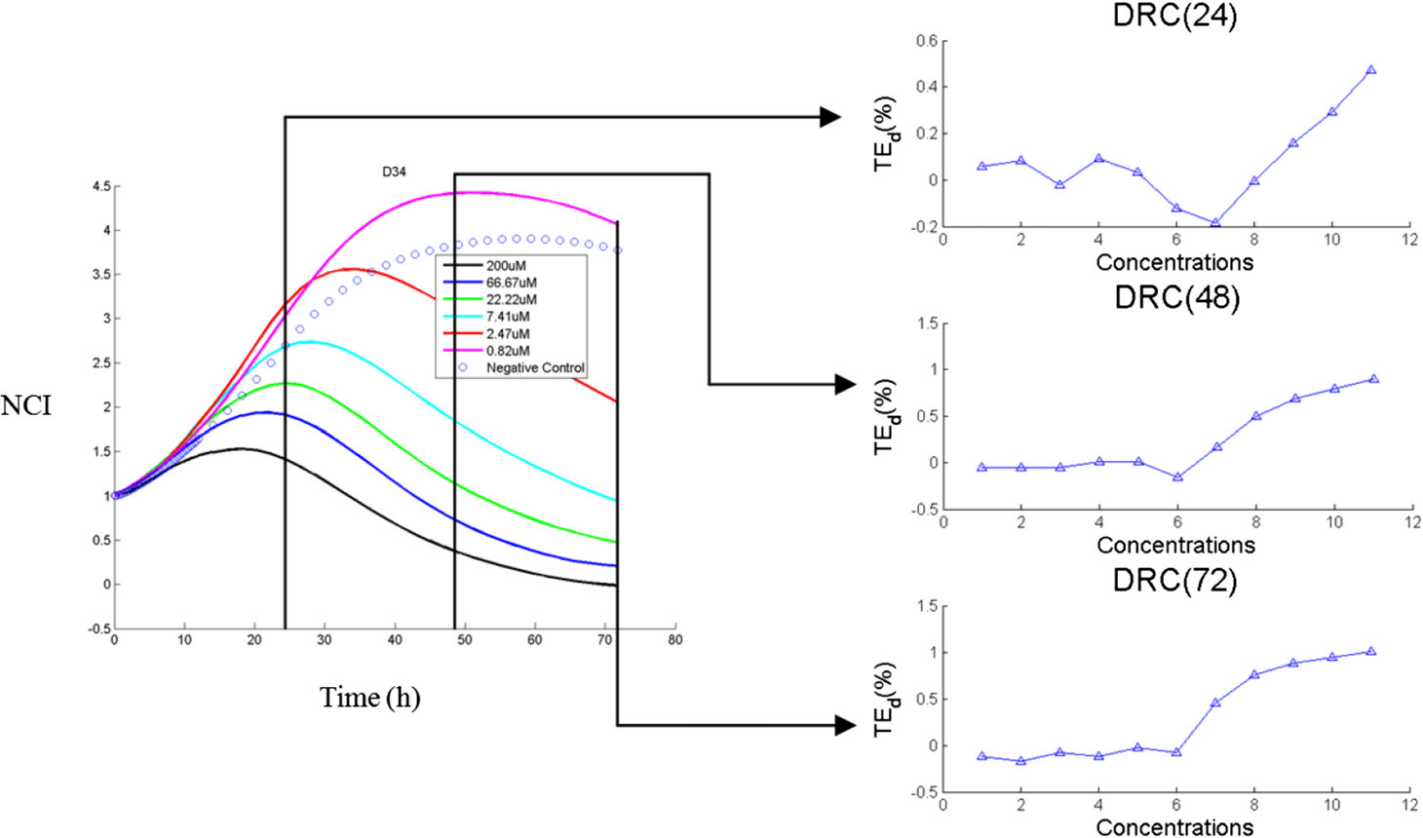
DRC of 5-FU from TCRCs

From (5), and using the *TE*_*t*_ defined by (3), we can define DRC at any given time *t* in our data set. Taking the compound 5-FU in cluster 1 as an example, we construct the DRC at 24 h, 48 h and 72 h as shown in Fig. 11.

**Fig. 12:**
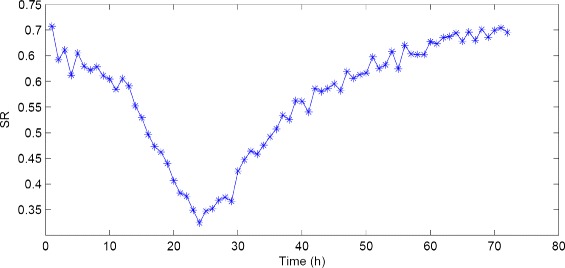
SVM SR distribution for two-cluster C1 and C10 using DRC(*t*)

According to the definition of DRCs in (5), DRC contains information regarding the reaction of the cell growth to the increment of the chemical concentrations. It is thus reasonable to assume that the compounds having different MOA may trigger different concentration-related reactions. Consequently, using DRCs data as training set may offer a way to improve the classification SR for those data that are not easy to be classified using TCRCs as input. Based on this approach, we carry out a SVM binary classification for C1 and C10 using DRC as input at a specified time point, and then linking the results at different time points together. The SVM results for clustering C1 and C10 are reported in Fig. 12. Different from using 803 data in TCRC(11), only 11 data are taken as input using DRC at a given time. The computational time is faster than that required based on TCRC as input data, but the overall SR is obviously not as good as those using TCRC. However, the plot in Fig. 12 reveals useful information, namely the time interval leading to a better SR can be determined. Thus, the methodology may offer a possible way to improve the performance of machine learning algorithm for imbalanced data set, since the time interval corresponding to low SR can be discarded in the input data.

**Fig. 13:**
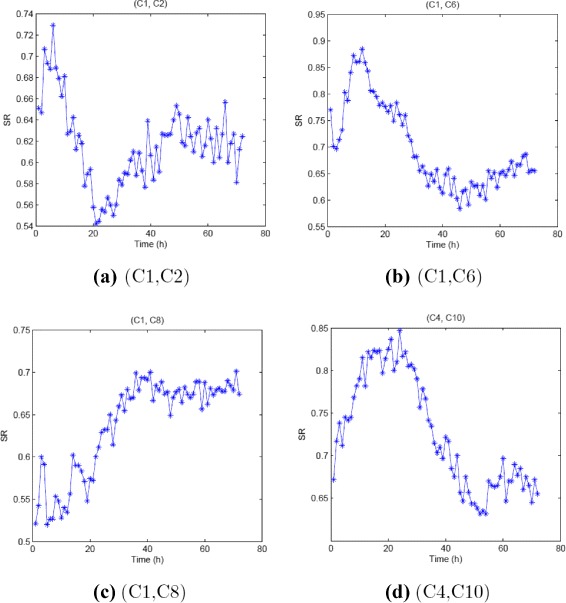
SR distribution for (**a**) (C1, C2), (**b**) (C1, C6), (**c**) (C1, C8) and (**d**) (C4, C10)

Figure 13 displays the SR for binary clustering (C1, C2), (C1, C6), (C1, C8), (C4,C10). Recall that the four cases represent typical imbalanced data, and poor SR is observed using TCRC(11) as input as reported in Table 5. In Table 8, appropriate time intervals are selected by ignoring the time intervals corresponding to low SR. Using the TCRC(11) selected at the specified time intervals, the SR using SVM applied to RCRC(11) and W_5_(4) are reported in Table 9. Using the selected TCRC at certain time interval for cases with imbalanced data, the SVM SR is clearly improved for all cases as shown in Table 9. However, more work is needed to investigate the best way to utilize the information from DRC to further enhance the performance of SVM.

**Table 8:** Selected time interval for TCRCs

Two-cluster	(C1,C2)	(C1,C6)	(C1,C8)	(C4,C10)
Selected interval	30–72 h	1–30 h	25–72 h	1–40 h

**Table 9:** Improvement of SR by using TCRCs at selected time points from DRC distribution

	Time for 1–72 h		Selected time	
	TCRC	W_5_(4)	TCRC	W_5_(4)
(C1, C2)	0.734	0.742	0.736	0.797
(C1, C6)	0.809	0.779	0.830	0.857
(C1, C8)	0.699	0.720	0.803	0.766
(C4, C10)	0.695	0.795	0.716	0.832

## Conclusion

In this paper, we present an innovative approach using machine learning for toxicity assessment. The computational tools are developed based on ANN and SVM, which are capable of learning data from given TCRCs with known MOA clustering information and then making MOA classification for untested chemical compounds. There are two challenges and difficulties of this work. First the input data arising from the time-series TCRC data contains more than 850 data, and secondly, only limited data set are available for some clusters. A novel data processing technique using wavelet transform is introduced, so that not only a great reduction in input data is achieved but the MOA classification is more accurate due to wavelet coefficients have the ability to extract important features from the original TCRC data. Instead of using more than 850 data from the TCRCs with 11 concentrations, we only require 229 and 157 wavelet coefficients as input data to the developed data mining tools. In this study, it is also revealed that taking account the information from the negative control curve enhances the performance of the MOA classification. It has been illustrated that the machine learning algorithm can be improved by utilizing information from DRC, so that a time interval leading to higher classification success rate can be selected as input. From the computational simulations, SVM is more effective compared to ANN for MOA classification. The developed SVM classifier has been tested for multi-cluster MOA classification, and impressive SR in the range of 85 to 95 % is obtained for *m*-cluster classification where 2≤*m*≤4. The present work concludes that SVM is an effective and powerful machine learning tool for toxicity profiling.

It is noted that the proposed SVM is tested on the limited training and testing data, to perform a reliable validation of the proposed machine learning approach, it is desirable if more testing data are available. Even though the present study focuses on a MOA classification, the approach could be extended to other type of classifications such as a Globally Harmonized System (GHS) classification in toxicology investigation. Instead of a supervised learning approach, it is of great interest to consider an unsupervised methodology. Moreover, to better handle a multi-cluster classification and to enhance the robustness of a machine learning approach, it is useful to develop an expert system consisting of various classifiers, so that reliable classification results can be determined by incorporating a validation procedure.

## Additional file

Additional file 1 63 chemicals in 10 MOA clusters. This file includes the 63 compounds used for in the MOA classification. Details including the used solvent and concentrations are also provided. (PDF 19 kb)

## Abbreviations

CI: Cell index
DRC: Dose response curve
MOA: Mode of action
ANN: Artificial neural network
SR: Successful rate
RTCA: Real time cell analysis
SVM: Support vector machine
TCRC: Time-concentration response curve
