# Supplementary material for: Machine learning algorithms for mode-of-action classification in toxicity assessment
Source: BioData Min. 2016 May 13;9:19. doi: 10.1186/s13040-016-0098-0 (PMC4866020; doi:10.1186/s13040-016-0098-0)
Supplement: Additional file 1 — 63 chemicals in 10 MOA clusters. This file includes the 63 compounds used for in the MOA classification. Details including the used solvent and concentrations are also provided. (PDF 19 kb) [file 13040_2016_98_MOESM1_ESM.pdf]

Additional File: 63 chemicals in 10 MOA clusters

**Cluster I**: DNA/RNA-Nucleic Acid Targets	Solvent	Concentration(1:3)
5-Fluorouracil (5-FU)	DMSO	200 μM to 3.39 nM
Gemcitabine HCl	H_2O	1650 μM to 27.94 nM
Etoposide phosphate	DMSO	200 μM to 3.39 nM
Doxorubicin	H_2O	100 μM to 1.69 nM
Merbarone	DMSO	200 μM to 3.39 nM
Clofarabine	H_2O	25 μM to 0.42 nM
Hydroxyurea	H_2O	10 μM to 169 nM
SN-38	DMSO	200 μM to 3.39 nM
Topotecan	DMSO	95 μM to 1.61 nM
Irinotecan	DMSO	160 μM to 2.71 nM
Cytosine	H_2O	8950 μM to 151.57 nM
ABT-888	DMSO	308 μM to 5.22 nM
Mitoxantrone dihydrochloride	DMSO	150 μM to 2.54 nM
CRT0044876	DMSO	194 μM to 3.29 nM
NU7026	DMSO	20 μM to 0.34 nM
Mitomycin C	DMSO	200 μM to 3.39 nM
Cordycepin	DMSO	200 μM to 3.39 nM
Actinomycin D	DMSO	2 μM to 0.0339 nM
Cisplatin	H_2O	150 μM to 2.54 nM
Ochratoxin A	DMSO	10 μM to 0.17 nM
**Cluster II**: Transport Protein-Primary Activetransporter Targets	Solvent	Concentration(1:3)
Brefeldin A	DMSO	40 μM to 0.68 nM
Exo 1	DMSO	300 μM to 5.08 nM
Leptomycin B	DMSO	20 nM to 0.000339 nM
Concanamycin A	DMSO	0.2 μM to 0.003 nM
Thapsigargin	DMSO	2 μM to 0.0339 nM
BHQ	DMSO	400 μM to 7 nM
**Cluster III**: Protein-Actin Targets		
Bafilomycin A1	DMSO	0.3212 μM to 0.01 nM
Cytochalasin D	DMSO	20 μM to 0.339 nM
Cytochalasin B	DMSO	20 μM to 0.339 nM
Latrunculin A	DMSO	2 μM to 0.0339 nM
Latrunculin B	DMSO	2 μM to 0.0339 nM
**Cluster IV**: Protein-Tubulin Targets		
Docetaxel	DMSO	1 μM to 0.02 nM
Paclitaxel	DMSO	20 μM to 0.339 nM
Vincristine Sulfate	H_2O	250 μM to 4.23 nM
Vinblastine sulfate	H_2O	40 μM to 0.68 nM
**Cluster V**: Ribosome-50S Subunit Targets		
Emetine	H_2O	50 μM to 0.847 nM
Puromycin	H_2O	1000 μM to 17 nM
Anisomycin	H_2O	10 μM to 0.17 nM
**Cluster VI**: Transport Proteins-Electrochemical Potential-driven Transporters		
Oligomycin	DMSO	20 μM to 0.339 nM
Antimycin A	DMSO	200 μM to 3.387 nM
Rotenone	DMSO	200 μM to 3.387 nM
CCCP	DMSO	100 μM to 1.69 nM

**Cluster VII**: Ion Channel Targets	Solvent	Concentration(1:3)
Valproic acid	H_2O	50 mM to 847 nM
BAPT-am	DMSO	60 μM to 1 nM
**Cluster VIII**: Enzyme Targets		
Cyclosporin A	DMSO	100 μM to 1.69 nM
FK-506	DMSO	50 μM to 1 nM
(S)-HDAC-42	DMSO	128 μM to 2.17 nM
SAHA	DMSO	151 μM to 2.56 nM
W7 HCl	DMSO	200 μM to 3.39 nM
**Cluster IX**: Receptors		
benzo[a]pyrene	DMSO	100μM to 1.69 nM
**Cluster X**: Protein- Motor Targets		
Monastrol	DMSO	100 μM to 1.69 nM
S-trityl-cysteine	DMSO	100 μM to 1.69 nM
Dimethylenastron	DMSO	40 μM to 0.68 nM
Y-27632	DMSO	188 μM to 3.18 nM
HA1100 hydrochloride	H_2O	1000μM to 16.94 nM
Ro32-3555	DMSO	200 μM to 3.39 nM
Batimastat	DMSO	200 μM to 3.39 nM
MLCKInhibPep18	H_2O	94.5 μM to 1.6 nM
Blebbistatin	DMSO	100 μM to 1.69 nM
ML7 hydrochloride	DMSO	100 μM to 1.69 nM
FAKInhibitor14	H_2O	2500 μM to 42.34 nM
PF573228	DMSO	40 μM to 0.68 nM
PF431396	DMSO	5 μM to 0.08 nM
